# Environmental correlates and fine-scale distribution of *Amblyomma americanum*, *Ehrlichia* spp., and *Rickettsia amblyommatis* at a single site in south-central Virginia

**DOI:** 10.1186/s13071-025-06999-2

**Published:** 2025-09-29

**Authors:** Dayvion R. Adams, Roland Kays, Michael H. Reiskind

**Affiliations:** 1 Department of Entomology and Plant Pathology, North Carolina State University, 100 Derieux Place, 2319 Gardner Hall, Raleigh, NC 27607 USA; 2 North Carolina Museum of Natural Sciences, Raleigh, NC USA; 3 Department of Forestry and Environmental Resources, North Carolina State University, Raleigh, NC USA

## Abstract

**Background:**

As tick-borne disease cases surge in the southeastern United States, there is a growing need to understand the ecological risk factors and distribution of the most abundant tick vector, *Amblyomma americanum*. While previous research has examined ecological factors influencing other tick vectors, such as *Ixodes scapularis*, few studies have evaluated micro-landscape variables associated with *A. americanum* abundance. Moreover, the spatial distribution of tick vectors is rarely studied at biologically relevant, fine-scale resolutions (i.e., < 100 m).

**Methods:**

In this study, we applied a 5-m^2^ sampling grid to a field site with diverse habitat structure to identify the micro-landscape variables associated with adult and nymphal abundance of *A. americanum* using linear modeling approaches. We also characterized the spatial distribution patterns of both life stages across our field site using hotspot analyses. Lastly, we applied the same hotspot analyses to ticks infected with *Ehrlichia* spp. and *Rickettsia amblyommatis*, two microbial agents associated with *A. americanum* in nature.

**Results:**

We found that different landscape variables at field site influenced adult and nymphal abundance of *A. americanum*, with edge habitat emerging as a significant predictor for both life stages. While adults were broadly distributed across habitat types, nymphs were more spatially restricted to forested areas, aligning with previous observations of nymphal distribution patterns.

**Conclusions:**

The significance of edge habitat and forest variables on *A. americanum* abundance highlights promising targets for control interventions and can be used to develop public health recommendations to reduce tick encounter risk and subsequent pathogen transmission.

**Graphical Abstract:**

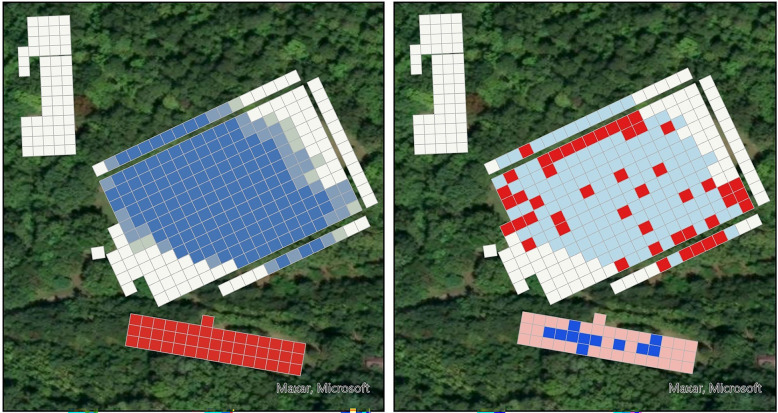

**Supplementary Information:**

The online version contains supplementary material available at 10.1186/s13071-025-06999-2.

## Background

Tick-borne diseases (TBDs) are increasing steadily in the United States, accounting for 75% of all arthropod-borne diseases [1]. While changes in climate and landscape are widely recognized as contributing factors, the extent to which these factors influence pathogen transmission remains unclear [2–4]. For instance, reforestation across much of the eastern USA, combined with the elimination of natural predators, has led to a significant resurgence of white-tailed deer (*Odocoileus virginianus,* Zimmermann), which in turn has contributed to the proliferation of blacklegged (*Ixodes scapularis*, Say) and lone star (*Amblyomma americanum,* Linnaeus) ticks [5]. Deer serve as important propagation, reservoir, and dispersal hosts for these ticks [5, 6], and in many areas of the eastern USA, overabundant deer populations facilitate the spread of ticks and their associated pathogens across the landscape [7]. The reestablishment of deer exemplifies how broad ecological changes, such as reforestation and altered predator–prey dynamics, are reshaping tick distributions and TBD risk, especially in the southeastern USA.

While *I. scapularis* has historically received the most research attention, recent studies have increasingly highlighted the importance of *A. americanum* as a vector of human and animal pathogens, including multiple *Ehrlichia* species that cause ehrlichiosis [8, 9], Bourbon virus [10], and Heartland virus [11], and as the cause of alpha-gal syndrome in humans (formerly known as red meat allergy) [12]. Additionally, ongoing research aims to clarify the public health implications of the widespread endosymbiont *Rickettsia amblyommatis*, which may contribute to human disease but remains poorly understood [13]. In the southeastern USA, *A. americanum* is currently the tick most frequently encountered by people, particularly in forested areas with abundant deer populations [14–16]. Increasing suburban development within tick habitats and growing participation in outdoor recreation both heighten human exposure and facilitate pathogen transmission [15, 17]. However, the specific micro-landscape factors (e.g., vegetation length, forest cover, humidity) that contribute to *A. americanum* exposure and subsequent pathogen transmission remain poorly understood. This highlights the need for further research to understand how landscape characteristics and modifications influence tick distributions and consequent TBD risk.

Extensive research has explored large-scale TBD transmission patterns in the eastern and Midwest USA [2, 3, 17, 18]. Large-scale studies are valuable for understanding and forecasting disease spread and for disseminating public health information. For example, Diuk-Wasser et al. [3] used large-scale tick collections and landscape assessments to understand the role of habitat fragmentation in Lyme disease transmission in a high-incidence area of New York. However, such studies often fail to identify specific environmental predictors of tick presence, as ticks and their vertebrate hosts interact with the environment at much finer, meter-level spatial scales. Although less common, several studies have investigated fine-scale (< 200 m) environmental factors that influence tick abundance, focusing on *I. scapularis* and related vectors of Lyme disease [19–22]. For example, research in urban environments has identified the presence of log piles in residential yards, at a scale of < 100 m, as a key predictor of tick abundance due to its role in providing refuge for tick-carrying rodents [23]. Another study by Dumas et al. [22] used a micro-landscape approach to identify areas of high Lyme disease transmission risk in a Canadian recreational park. Based on their findings, the authors recommend specific interventions to reduce human–tick contact on high-risk recreational trails, such as enhanced landscaping (e.g., vegetation trimming). Studies like these exemplify how identifying fine-scale landscape characteristics can help predict tick abundance and inform potential tick management recommendations.

Despite the ongoing public health threat posed by *A. americanum*-pathogen transmission in the southeastern USA, only a few studies have investigated the environmental variables influencing its abundance. These studies have identified forest-related variables such as carbon content in leaf litter and forest cover as key predictors of *A. americanum* abundance [14, 24, 25], yet none have evaluated the fine-scale distribution of *A. americanum*. Fine scales (< 200 m) are biologically relevant to *A. americanum* due to their limited movement range, as demonstrated by Marshall et al. [26] using mark–recapture methods, which revealed that individuals only moved up to 9 m/day in nature, with an average of 3.2 m. In contrast, common vertebrate hosts can move over relatively larger spatial scales. For example, white-tailed deer have been reported to travel daily distances of up to several thousand meters within their home ranges, while mammalian carnivores may cover even greater distances, up to 13,000 m/day [27–29]. Although the movement of vertebrate hosts facilitates widespread dispersion across the environment, it is the fine-scale movement of ticks that is most relevant to pathogen transmission to humans. Understanding fine-scale distributions of this species, together with previous behavioral studies, will improve assessments of tick bite risk and aid in the development of targeted control strategies at this scale.

In this study, we investigated fine-scale landscape variables within one field site characterized by diverse habitat structures to better understand the spatial distribution and microclimatic preferences of *A. americanum* across life stages. By characterizing environmental features at the 5-m^2^ sampling squares scale, we tested multiple hypotheses derived from previous tick ecology studies about the role of environmental factors influencing tick abundance, such as habitat edges [3, 30] and vegetation length, managed through mowing [31]. We further expanded this analysis by mapping tick and pathogen distributions at a 5-m^2^ scale, allowing us to examine spatial patterns that have not been previously documented for this tick–pathogen system. The results of this study may be extrapolated to broader spatial scales to assess whether these patterns remain consistent at larger scales. We hypothesized that adult and nymphal *A. americanum* would exhibit distinct, non-random spatial patterns related to specific environmental variables, and that pathogen hotspots would correspond to areas with high tick densities, particularly along habitat edges. Through this dual approach, combining micro-landscape assessment with high-resolution spatial analysis, we aim to provide new insights into the ecology and public health relevance of *A. americanum* in the southeastern USA. Rising cases of ehrlichiosis and spotted fever group rickettsioses underscore the urgent need for localized, scale-appropriate strategies for tick surveillance and management.

## Methods

### Study site

The study site is a 67-acre private property in Red Oak, Virginia, approximately 32 km from the North Carolina border (Supplementary Figure 1) [32]. The site is within the Piedmont region of Virginia, which is characterized by hot, humid summers and mild winters. Our sampling period coincided with the time of year that typically receives the most precipitation. The property is heterogeneous, with natural, heavily vegetated areas and routinely mowed areas. To evaluate landscape risk factors influencing *A. americanum* abundance, we stratified our sampling across four distinct areas of the property, herein referred to as habitat plots, each representing unique landscape characteristics: (1) a forested area dominated by white oak (*Quercus alba*), pines (*Pinus* spp.), hickories (*Carya glabra*), and a bushy understory of various shrub species; (2) an open field with long and medium grass (described below); (3) an open field with short and medium grass (described below); and (4) a domicile area consisting of a mix of unpaved gravel, forested underbrush, open-canopy grass patches, and ornamental vegetation. The domicile buildings were not used as primary residences and were only visited by the property owners once or twice a month. These structures included a storage shed, a bunkhouse, and an outhouse. The study site contained a diverse vertebrate community with evidence of black bears (*Ursus americanus*, Linnaeus), white-tailed deer, groundhogs (*Marmota monax*, Linnaeus), eastern cottontails (*Sylvilagus floridanus*, Bachman), and wild turkeys (*Meleagris gallopavo*, Linnaeus). In addition, several semi-ground-dwelling bird species were observed at the site, including northern cardinals (*Cardinalis cardinalis*, Linnaeus), turkey vultures (*Cathartes aura*, Linnaeus), and American crows (*Corvus brachyrhynchos*, Brehm), all of which may serve as hosts for ticks.

### Field sampling design

We sampled the study site over two consecutive years. In year 1, all sampling was conducted over 10 days, from 19 June to 17 July 2023. In year 2, all sampling was conducted over 8 days from 27 June to 16 July 2024. All tick sampling was conducted between 10 a.m. and 2 p.m., the period when tick activity is expected to be highest [33]. We avoided days with excessive rain that would hinder cloth sampling methods for ticks (i.e., drags and flags). At each habitat plot, we established a 5-m^2^ sampling grid using wooden stakes (Supplementary Figure 2). This sampling scale was selected based on the limited host-seeking movement of adult ticks, as a previous study found that they move an average of 3.2 m/day [26]. We collected spatial coordinates (i.e., longitude, latitude) using a Garmin 66st (Garmin Ltd., Olathe, KS, USA), which has reported accuracy of up to 2.5 m. The number of squares varied by habitat plot, with the short grass field (SGF) having the largest sampling area at 224 squares, followed by the long grass field (LGF) with 46 squares, and the domicile with 38 squares. The number of squares sampled in the forested plot varied between years: 11 squares were sampled in year 1, while 59 squares were sampled in year 2. We expanded forest sampling in year 2 to increase sampling effort in this plot and to assess potential edge effects observed during the previous year. In year 1, each square was sampled four times, twice by dragging and twice by flagging. The only exception was in the forest, where only two dragging events were conducted because flagging was not feasible. In year 2, all squares, including those in the forest, were sampled once by dragging and once by flagging. Sampling for a given square was never conducted on the same day, with at least one full day, often more, between resampling events to avoid bias. For each sampling event, we recorded the number of times a square had been sampled and the sampling order to identify potential biases in tick collection.

Drag cloths were made of 1-m^2^ cotton flannel cloth attached to a wooden dowel with a diameter of 3 cm, with two 1-m sections of rope attached to a polyvinyl chloride (PVC) pipe used as a handle, following Centers for Disease Control and Prevention guidelines [34]. We conducted drag sampling by dragging the entire 5-m^2^ grid square. At the end of each square, we checked the bottom and top of the drag cloth for the presence or absence of ticks, collecting and recording any. Flagging involved sweeping the flag cloth across the square, checking for ticks on both sides of the flag cloth every 1 m^2^. Flag cloths were made of 1-m^2^ cotton flannel cloth attached to a wooden pole [34]. All collected ticks were preserved in 70% ethanol and stored at −20 °C. Adult ticks were stored individually in tubes, while nymphs and larvae were pooled by grid square, with up to 25 nymphs per pool; multiple tubes were used if necessary for a single square. All ticks were identified to species using published morphological keys, except for *Ixodes* spp. larvae, which we only identified to genus [35–40].

To evaluate landscape risk factors contributing to *A. americanum* abundance, we recorded the following characteristics: habitat plot type sampled (SGF, LGF, forest, domicile), vegetation length, presence of leaf litter, presence of rocks on unpaved roads, adjacency to human structures (e.g., buildings), planted ornamental vegetation, mowed vegetation, sparse vegetation (e.g., patches of vegetation in a heterogeneous square), shade, exposed soil, adjacency to forests (e.g., forest edge), adjacency (e.g., < 5 m) to human-made roads (e.g., road edge), adjacency to open fields (e.g., field edge), dried or desiccated vegetation, and proximity to a water source (Supplementary Table 1). We selected these characteristics because they are biologically relevant factors that may influence tick abundance and serve as potential targets for intervention in tick control. We categorized vegetation length into three groups: 0–4 cm (short vegetation), 5–30 cm (medium vegetation), and 31 cm or longer (long vegetation). Although vegetation was not identified to species level, the site supported a diverse and heterogeneous plant community. We recorded the number of times each square was sampled to assess potential oversampling effects. The following model predictors were treated as either categorical or continuous variables: habitat plot, humidity, temperature, sampling method, year, and sample order (Supplementary Table 1). All other predictors in the tested models were binary (i.e., presence/absence). We recorded temperature and humidity using iButtons (iButton Link Technology, Whitewater, WI, USA) every 15 min, which were attached inside 20 plastic cups, which were inverted and placed in squares within our habitat plots. Four cups, one in each habitat plot, were placed in a randomly selected square and remained in place for the entire temperature and humidity data collection period. The remaining 16 cups were rotated weekly to randomly selected squares, with the number of cups in each habitat plot proportional to the number of squares sampled in that plot, over 3 weeks. This resulted in a total of 18,144 readings for the SGF, 6048 readings for the LGF, 10,080 readings for the forested habitat, and 6048 readings for the domicile habitat. This approach yielded average temperature and humidity readings for each habitat plot over the sampling period. The corresponding habitat plot values were assigned to each grid square for each 15-min sampling interval.

### Molecular diagnostics

We extracted DNA from all *A. americanum* adults and nymphs to screen for *R. amblyommatis*, *Ehrlichia chaffeensis*, and *E. ewingii*, as these microbial agents are known to be associated with *A. americanum*. We completed extractions using Qiagen DNeasy Blood and Tissue Kit (Qiagen, Hilden, Germany) according to the manufacturer’s recommendations with slight modifications, described below. We bisected all adult ticks longitudinally, using one half for extraction, while the other half was stored in 70% ethanol in case of extraction failure. Unlike adults, we extracted DNA from pools of up to 25 nymphs. To increase DNA yield, we incubated samples overnight in proteinase K and macerated ticks with a pestle on the second day of extraction. We stored extracted DNA at −20 °C using 60 µL of elution buffer (Qiagen, Hilden, Germany) until real-time polymerase chain reaction (qPCR) screening. To prevent contamination, we (1) sanitized materials between tick samples, (2) used new pestles for each tick maceration, and (3) completed extractions in separate areas from qPCR assays. For both *E. ewingii* and *E. chaffeensis* qPCR assays, we used the same forward primer, ECH16S-1 [41]. For *E. ewingii*, we used the EEW16S-97 reverse primer and EEW16S-P probe [42]. For *E. chaffeensis*, we used the reverse ECH16S-97 primer and ECH16S-38 probe [41]. For *R. amblyommatis* detection, we used Ra477F and Ra618R primers, with the Ra532P probe [43]. All probes in our study utilized the FAM (6-carboxyfluorescein) fluorescent dye for DNA quantification. We completed all qPCR assays on a CFX Connect real-time PCR detection system (Bio-Rad, Hercules, CA, USA). We included positive and negative controls in all DNA extractions and qPCR assays to validate our results.

### Autocorrelation analysis

All spatial maps and autocorrelation analyses were completed in ArcGIS Pro (Esri, Redlands, CA, USA; version 3.4). To test our hypothesis of spatial autocorrelation in *A. americanum* abundance, we used both the optimized hotspot analysis and outlier analyses. The optimized hotspot analysis uses the Getis-Ord Gi* statistic to identify statistically significant clusters of grid squares with high tick abundance [44]. The optimized outlier analysis is similar to hotspot analysis but uses the Anselin local Moran's *I* statistic to identify hot and cold spots, as well as significant spatial outliers [45]. Analyses were conducted separately for adults and nymphs. In addition to analyzing tick abundance, we also conducted these analyses using tick pathogen data to test the hypothesis that these bacteria also exhibit spatial autocorrelation at fine scales. Due to smaller individual sample sizes, we grouped *E. ewingii* and *E. chaffeensis*-positive ticks for spatial analyses as *Ehrlichia* spp.

### Statistical modeling and analysis

We used a generalized linear mixed model (GLMM) approach to assess the landscape characteristics influencing *A. americanum* abundance. Our data best fit a negative binomial distribution with a log link function due to the frequency of zero collection events among the sampled squares. We first assessed the grid characteristic variables for collinearity and removed any variables that showed significant collinearity, defined as a correlation coefficient > 0.60. Using the remaining variables, we performed a stepwise variable removal to identify the best-fit model. We evaluated both year and sampling day as random effects, but year fit better as a fixed effect and was retained as an explanatory variable. To compare and assess the impacts of our explanatory variables, we scaled and zero-centered our covariates (i.e., standardized). Like our spatial analyses, we created models for both adults and nymphs. Additionally, due to significant spatial autocorrelation observed in our results, we separately tested models that included an autocorrelation explanatory variable. This was based on Moran’s *I* statistic from our localized hotspot analysis. Due to the strong influence of outliers on early nymph models, we applied a threshold of 10 nymphs per collection event, capping any values above 10. This cutoff was chosen based on both biological and statistical rationale. Biologically, collection events exceeding 10 nymphs were rare and often suggested unusual local conditions, such as repeated vertebrate host activity at the same grid square. Statistically, 10 nymphs represented the 99th percentile of our data (*n* = 29 collections), indicating that values above this point were extreme and not representative of typical collection events. Given the significance of edge habitat variables in both models, we conducted a post hoc Chi-square test of independence between edge status and infection status for both *Ehrlichia* spp. and *R. amblyommatis* to assess whether edge habitat was associated with tick infections. We conducted all data analysis using R statistical computing software (R Foundation for Statistical Computing, Vienna, Austria; version 4.3.2) and utilized the glmmTMB package for our modeling [46]. We used an alpha value of *P* = 0.05 for judging statistical significance.

## Results

**Fig. 1:**
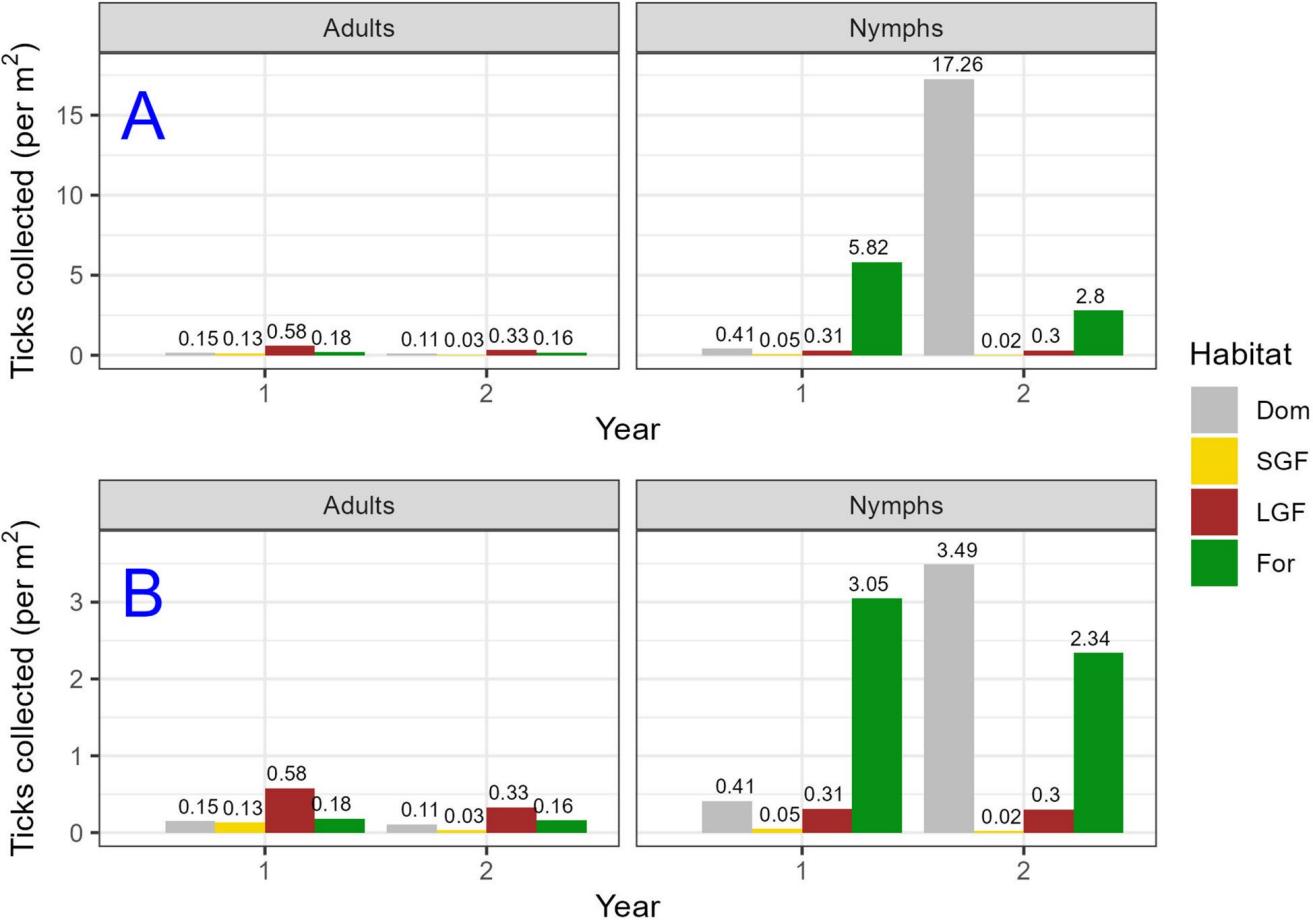
Mean *A. americanum* adult and nymph collection densities by year and habitat type **A** including outlier collections and **B** with outlier collections removed. Means were calculated by dividing the total number of ticks collected in each habitat by the total number of squares sampled, accounting for differences in sampling effort across habitat plots. Sampling effort was calculated as the number of squares sampled within a habitat plot and sampling year. Figure legend abbreviations are as follows: Dom: domicile, SGF: short grass field, LGF: long grass field, and For: forest

In total, we collected 2873 ticks across 1944 5-m^2^ squares, with substantial variation across sites and years (Fig. 1). In year 1, we collected 666 *A. americanum* (218 adults, 275 nymphs, 173 larvae), 25 adult *Dermacentor variabilis* (Say), six *Ixodes affinis* (Neumann) (two nymphs, four adults), one adult *Amblyomma maculatum* (Koch), one adult *Haemaphysalis longicornis* (Neumann), one nymph *Haemaphysalis leporispalustris* (Packard), and 182 *Ixodes* spp. larvae. In year 2, we collected 1821 *A. americanum* (73 adults, 1695 nymphs, 53 larvae), 12 adult *D. variabilis*, and seven *I. affinis* (two adults, five nymphs). Our collections yielded 396 *A. americanum* (134 adults, 54 nymphs, 195 larvae) from the SGF, 173 (102 adults, 66 nymphs) from the LGF, 464 (24 adults, 475 nymphs, one larva) from the forest, and 1519 (31 adults, 1375 nymphs, nine larvae) from the domicile plot (Supplementary Table 2). Temperature and relative humidity varied throughout the day as expected, with the two field plots having the highest temperature and lowest humidity, followed by the domicile plot. In contrast, the forested plot had the lowest temperatures and highest humidity (Supplementary Figure 3).

Molecular analysis of microbial agents revealed a total of 15 (1.97%) *E. ewingii*-positive samples (Supplementary Table 3), 30 (3.95%) *E. chaffeensis*-positive samples (Supplementary Table 4), and 477 (62.85%) *R. amblyommatis*-positive samples (Supplementary Table 5). Five (1.04%) pooled nymph samples were co-infected with all three bacteria and were collected from the forested plot on the same day. Results by habitat plot showed that the domicile habitat had 13 (4.94%) samples positive for *Ehrlichia* spp., including two (5.88%) adults and 11 (4.80%) nymph pools, while 168 (66.14%) samples, consisting of 23 (67.65%) adults and 145 (63.32%) nymph pools, tested positive for *R. amblyommatis*. In the forested plot, 18 samples (9.49%) comprising 13 (50.0%) adults and five (4.54%) nymph pools were positive for *Ehrlichia* spp., and 102 (74.45%) samples, comprising 21 (80.77%) adults and 81 (73.63%) nymph pools, were positive for *R. amblyommatis*. At the SGF plot, seven (3.52%) samples (all adults) tested positive for *Ehrlichia* spp., and 126 (63.32%) samples, including 97 (67.83%) adults and 29 (51.79%) nymph pools, tested positive for *R. amblyommatis*. Similarly, the LGF site had seven (4.35%) positive samples for *Ehrlichia* spp. (all adults) and 84 (52.17%) positive samples for *R. amblyommatis*, comprising 52 (49.52%) adults and 32 (57.14%) nymph pools.

**Fig. 2:**
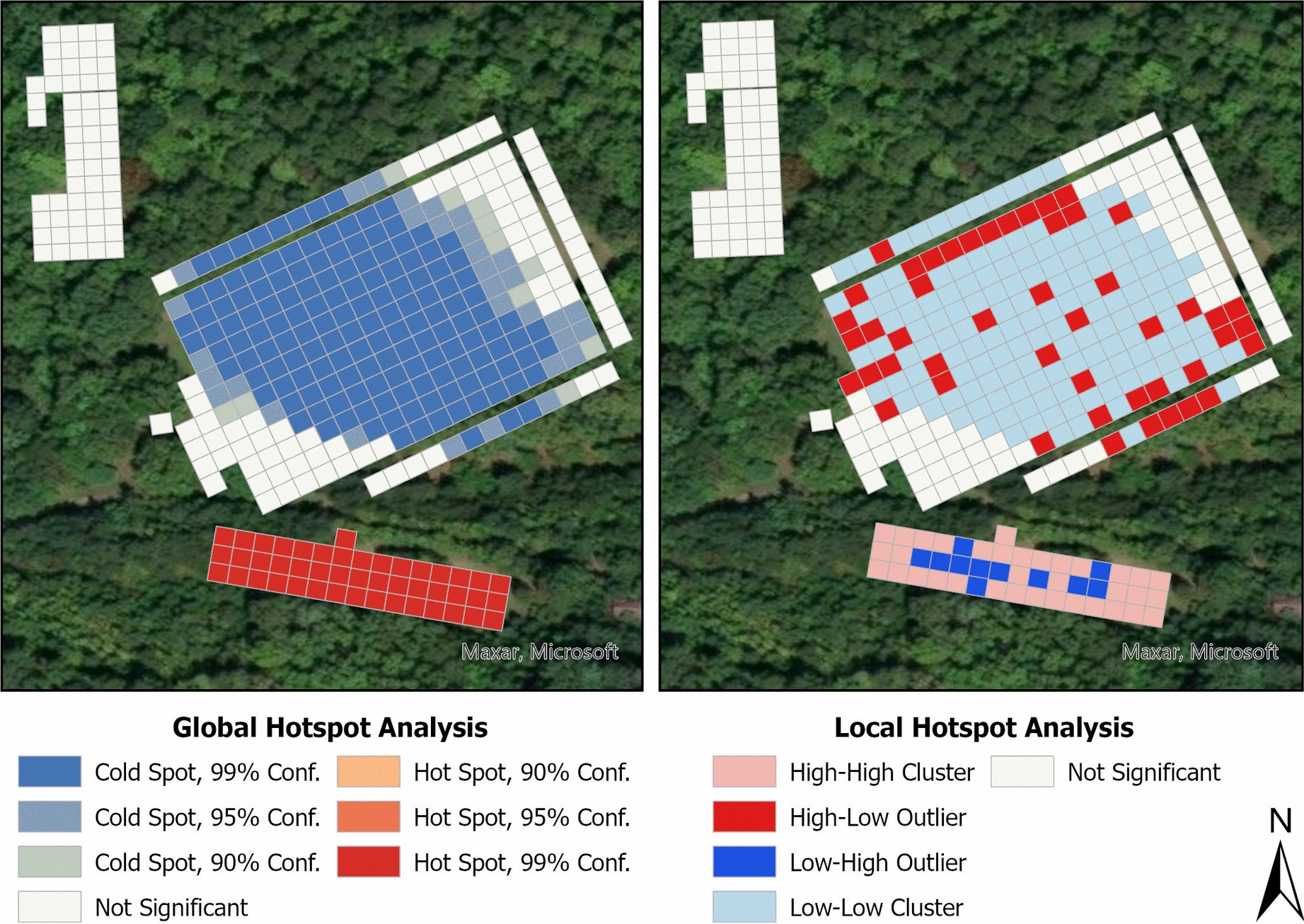
Results of spatial hotspot analyses for *A. americanum* adult counts at the field sampling site. Grid squares represent 5-m^2^ areas. Global spatial clusters (left) were identified using optimized hotspot analysis with the Getis-Ord Gi* statistic, while local hotspots and outliers (right) were detected using Moran’s *I* spatial statistic. High–high clusters represent significant local hotspots where high-count squares are near other high-count squares, while low–low clusters represent significant local cold spots where low-count squares are near other low-count squares. High–low outliers represent squares with higher counts than expected based on surrounding low-count squares, while low–high outliers represent squares with lower counts than expected based on surrounding high-count squares

**Fig. 3:**
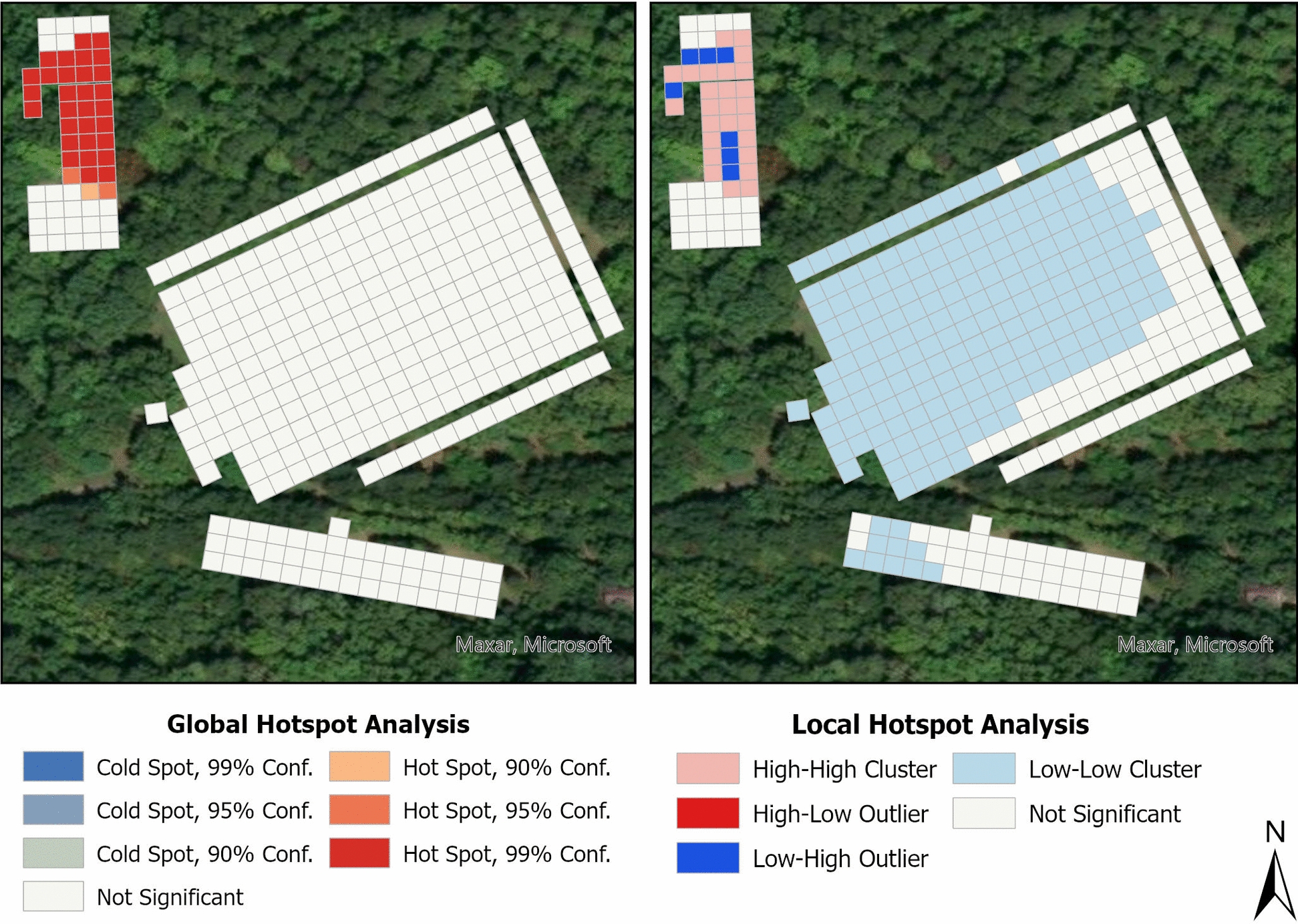
Results of spatial hotspot analyses for *A. americanum* nymph counts at the field sampling site. Grid squares represent 5-m^2^ areas. Global spatial clusters (left) were identified using optimized hotspot analysis with the Getis-Ord Gi* statistic, while local hotspots and outliers (right) were detected using Moran’s *I* spatial statistic. High–high clusters represent significant local hotspots where high-count squares are near other high-count squares, while low–low clusters represent significant local cold spots where low-count squares are near other low-count squares. High–low outliers represent squares with higher counts than expected based on surrounding low-count squares, while low–high outliers represent squares with lower counts than expected based on surrounding high-count squares

Adult *A. americanum* were primarily distributed in the SGF and LGF habitat plots, particularly along sampled habitat edges, with fewer individuals collected in the domicile and forested plots (Supplementary Figure 4). In contrast, nymphal *A. americanum* were more abundant in the forested plot, with fewer collected in the SGF and LGF plots (Supplementary Figure 5). Spatial autocorrelation of adults supported these observations across our sampling site, with a statistically significant global hotspot in the LGF and a cold spot in the SGF; however, no significant spatial patterns were observed in the domicile and forested plots (Fig. 2). Despite these trends, high–low outlier collections were detected within hotspot areas in our local hotspot analysis, particularly along field edges (Fig. 2). In contrast, nymphal *A. americanum* exhibited a statistically significant global hotspot identified in the domicile and forested plots (Fig. 3). Local hotspot analysis revealed significant cold spots in both field plots and along one edge of the forested plot (Fig. 3), with low–high outlier collections observed in both forested and domicile plots (Fig. 3).

Our initial collinearity assessment revealed strong correlations between several grid-level habitat characteristics. Specifically, leaf litter was strongly correlated with forested grids, while long vegetation was strongly correlated with the LGF plot. Due to these correlations, we excluded the habitat plot type (e.g., LGF or domicile) from our final models. All listed variables were significant predictors of adult abundance, except for the order of grid square sampling (Table 1). For nymphs, all variables were significant except for the presence of landscaping rocks, proximity to a water source, and exposed soil. The strongest predictors of adult abundance, in order of decreasing effect size, were proximity to a water source, our spatial autocorrelation variable, sampling year, adjacency to forest, sampling method, presence of sparse or uneven vegetation, landscaping rocks, tree shading, order of grid square sampling, temperature, and humidity (Table 1). All variables had a positive effect on adult tick abundance, except for sampling year, presence of landscaping rocks, and order of sampling, which were associated with decreased abundance (Table 1). The strongest predictors for our nymph model, in order of decreasing effect size, were presence of leaf litter, adjacency to forest, presence of landscaping rocks, tree shading, proximity to a water source, sampling year, exposed soil, and our spatial autocorrelation variable (Table 1). All variables were positively associated with nymph abundance, except for exposed soil, which had a negative effect (Table 1). Our post hoc analysis revealed a significant association between edge habitat and infection status for both *Ehrlichia* spp. (*χ*^2^ = 6.80, *df* = 1, *P* = 0.009) and *R. amblyommatis* (*χ*^2^ = 80.74, *df* = 1, *P* < 0.001).

**Table 1:** Output of best-fit spatial GLMM models for *A. americanum* adults and nymphs, highlighting associated environmental variables influencing tick densities

Adults					Nymphs				
Covariate	Est.	SE (±)	*z*-value	*P*-value	Covariate	Est.	SE (±)	*z*-value	*P*-value
Field edge (Y)	0.391	0.391	3.160	0.002*	Intercept	−3.631	0.240	−15.120	< 0.001*
Water source (Y)	0.343	0.343	3.032	0.002*	Leaf litter	1.550	0.214	7.255	< 0.001*
Spatial autocorrelation	0.204	0.204	8.738	< 0.001*	Forest edge (Y)	1.277	0.183	6.990	< 0.001*
Year number (2)	−0.161	0.029	−5.505	< 0.001*	Rocks (Y)	1.027	0.264	3.887	< 0.001*
Forest edge (Y)	0.153	0.153	6.067	< 0.001*	Shaded (Y)	0.824	0.199	4.145	< 0.001*
Sampling method (flag)	0.134	0.026	5.103	< 0.001*	Water source (Y)	0.810	0.415	1.949	0.051
Sparse/uneven veg (Y)	0.132	0.132	3.701	< 0.001*	Year number (2)	0.554	0.165	3.369	< 0.001*
Rocks (Y)	−0.113	−0.113	−2.719	0.007*	Dirt floor (Y)	−0.544	0.303	−1.793	0.073
Intercept	0.105	0.050	2.099	0.036*	Spatial autocorrelation	0.265	0.040	6.604	< 0.001*
Shaded (Y)	0.063	0.063	2.176	0.030*					
Sample order	−0.031	0.017	−1.819	0.069					
Temperature	0.022	0.022	2.184	0.029*					
Humidity	0.009	0.009	2.017	0.044*					

Significant predictors are denoted with an asterisk next to the associated p-values. Comparison variables are shown in parentheses after each covariate, with “Y” representing “Yes” or 1 in binary coding. Covariates are ordered by the magnitude of their estimated coefficients

**Fig. 4:**
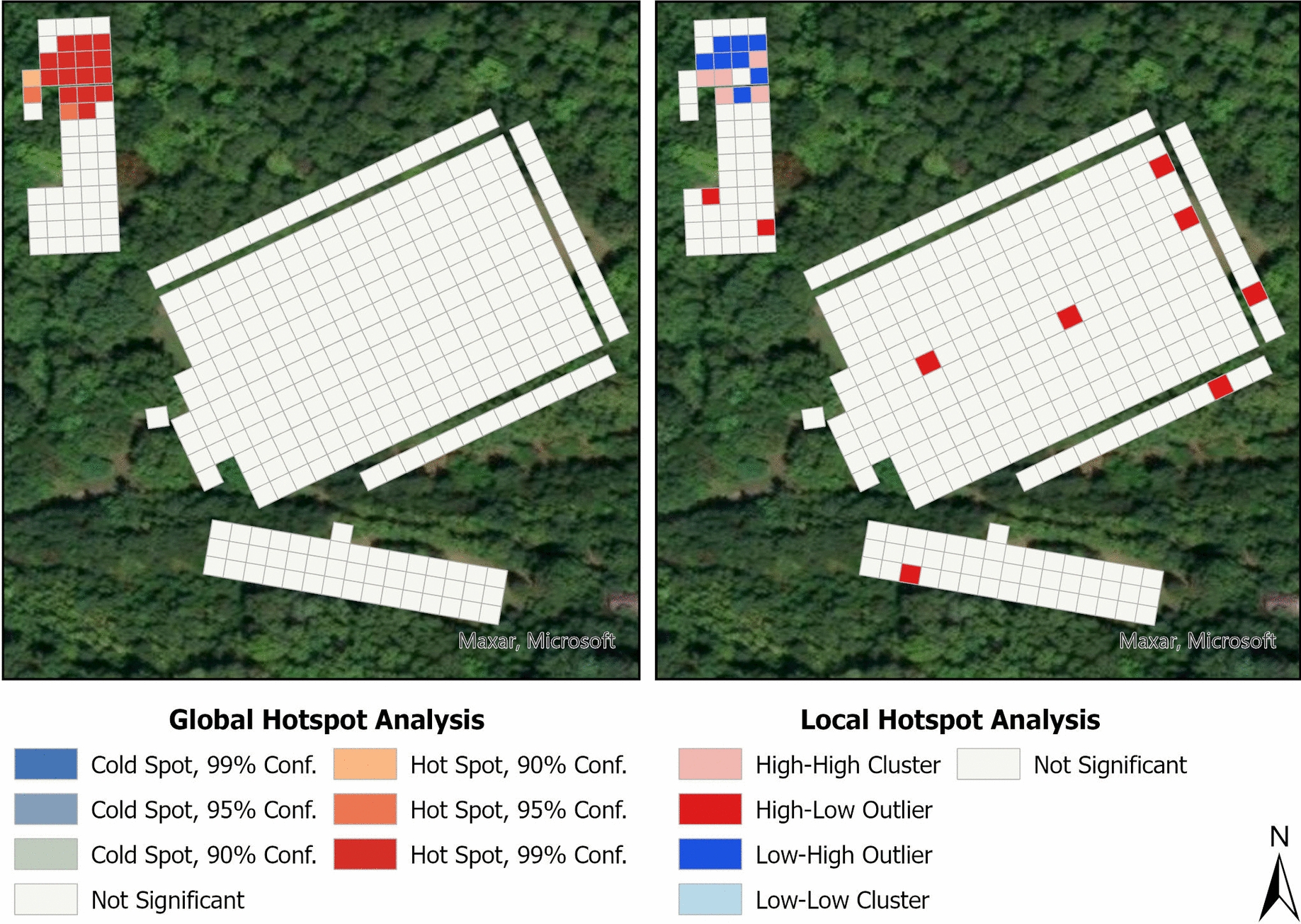
Results of spatial hotspot analyses for *Ehrlichia*-positive *A. americanum* sample counts at the field sampling site. Grid squares represent 5-m^2^ areas. Global spatial clusters (left) were identified using optimized hotspot analysis with the Getis-Ord Gi* statistic, while local hotspots and outliers (right) were detected using Moran’s *I* spatial statistic. High–high clusters represent significant local hotspots where high-count squares are near other high-count squares, while low–low clusters represent significant local cold spots where low-count squares are near other low-count squares. High–low outliers represent squares with higher counts than expected based on surrounding low-count squares, while low–high outliers represent squares with lower counts than expected based on surrounding high-count squares

**Fig. 5:**
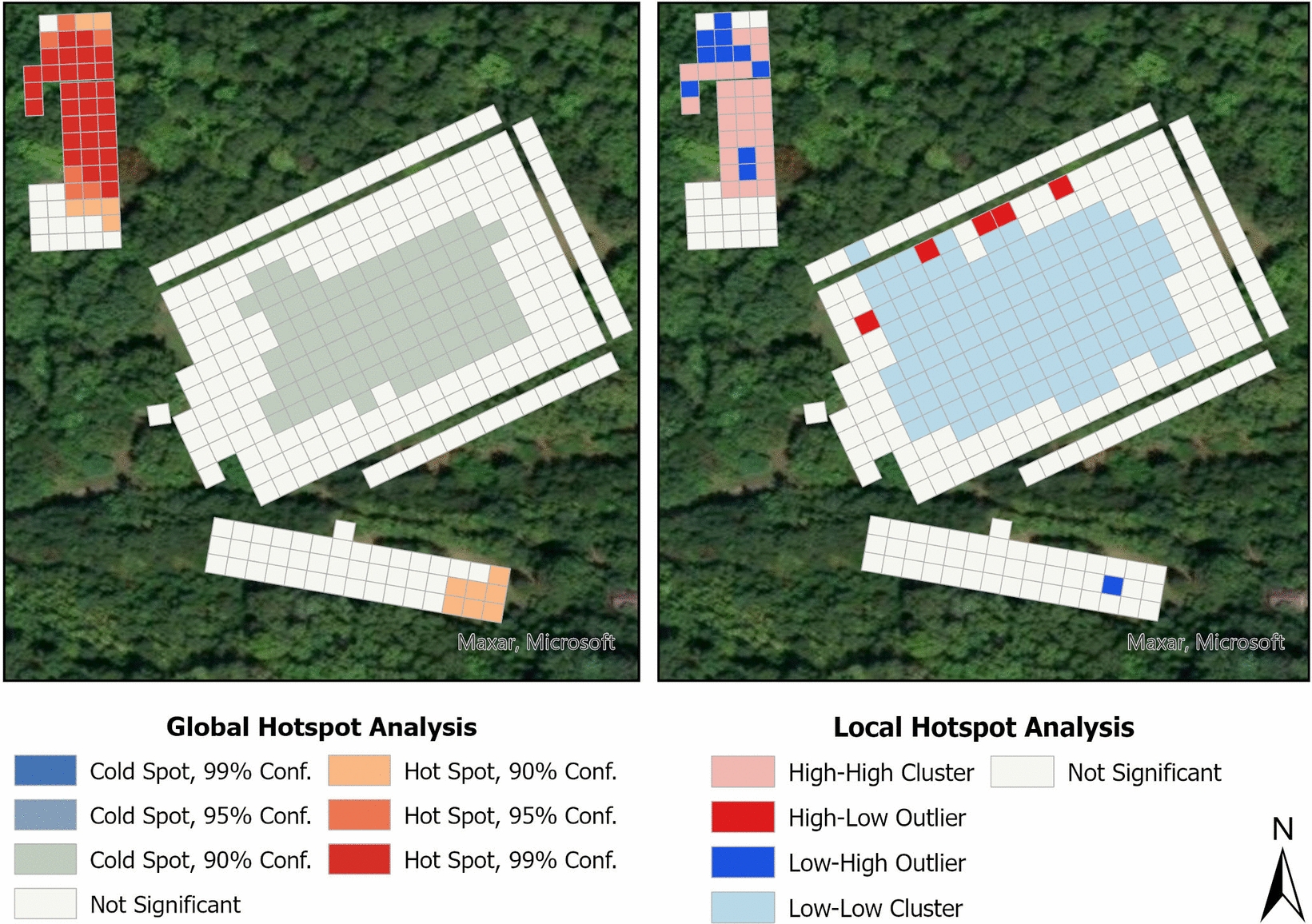
Results of spatial hotspot analyses for *R. amblyommatis*-infected *A. americanum* sample counts at the field sampling site. Grid squares represent 5-m^2^ areas. Global spatial clusters (left) were identified using optimized hotspot analysis with the Getis-Ord Gi* statistic, while local hotspots and outliers (right) were detected using Moran's *I* spatial statistic. High–high clusters represent significant local hotspots where high-count squares are near other high-count squares, while low–low clusters represent significant local cold spots where low-count squares are near other low-count squares. High–low outliers represent squares with higher counts than expected based on surrounding low-count squares, while low–high outliers represent squares with lower counts than expected based on surrounding high-count squares

*Ehrlichia* spp.-positive tick pools were distributed across all habitat plots, but were more concentrated in areas with high nymphal *A. americanum* abundance (i.e., forest, domicile) (Supplementary Figure 6). In contrast, the distribution of *R. amblyommatis* closely reflected the overall collection patterns of nymphal and adult ticks, with no single habitat plot showing a distinctly higher prevalence (Supplementary Figure 7). Statistically significant spatial autocorrelation was observed for *Ehrlichia* spp.-positive ticks, with hotspots in the forested and domicile plots and collection outliers distributed across habitat plots (Fig. 4). Similarly, *R. amblyommatis*-positive ticks exhibited significant spatial autocorrelation, with hotspots in the domicile and forested habitat plots, an additional hotspot in the LGF, a cold spot in the SGF, and outliers present in all habitat plots (Fig. 5).

## Discussion

As *A. americanum*-associated pathogens continue to rise in prevalence across the eastern USA, identifying the environmental risk factors contributing to this species’ abundance is crucial. Our study provides insights into the environmental variables influencing *A. americanum* adult and nymph presence at the meter scale, a level of analysis not previously conducted for the species. This scale is appropriate for tick distributions due to their limited mobility [26]. While several variables emerged as significant predictors of tick abundance, edge-related habitat features consistently stood out as important microhabitats for both adult and nymphal ticks, consistent with other tick fine-scale distribution studies [3, 30]. This finding highlights a potential avenue for targeted control of these resilient public health threats. Additionally, we characterize the spatial distributions for adult and nymph life stages and two microbial agents associated with this species and find that both bacteria demonstrate significant associations with habitat edges. Together, these findings enable us to provide meaningful public health recommendations on the factors affecting *A. americanum* distribution in the southeastern USA, where this species is particularly prevalent.

In our study, edge habitats emerged as strong predictors of tick abundance for both life stages, highlighting these areas as high-risk areas for encounters and pathogen transmission (Table 1). This finding aligns with research emphasizing the importance of edge habitats for other tick species, particularly forest edge ecotones [47, 48]. The accumulation of *A. americanum* at habitat edges suggests a biological mechanism that allows tick persistence in these microhabitats (Supplementary Figures 4 and 5). For example, consistent wildlife activity at habitat edges may influence tick deposition. Mammalian carnivores, known hosts of *A. americanum* [49], have been observed using human-made recreational trails [50]. This frequent use suggests that edge habitats may serve as travel routes, helping to explain the higher tick densities often observed in these areas. Alternatively, when dropping off vertebrate hosts, ticks may utilize edge habitats as suitable microhabitats for oviposition or molting. One limitation of our study is that we did not quantify edge size in nature, which may influence tick aggregation. Future work should aim to incorporate edge size measurements to refine our understanding of edge-associated risk. Edge habitats may offer favorable microclimates for tick survival relative to more open habitats, including cooler temperatures, higher relative humidity, and increased shading from potentially lethal direct sunlight. While we did not directly measure microclimatic differences between edge habitats and our habitat plots, we observed such differences among sampled plots (Supplementary Figure 3). Both the forested and domicile habitats exhibited these abiotic characteristics and had high tick abundance, supporting this hypothesis. Given the consistent rise in TBD transmission in the southeastern USA, these findings support advocating improved tick prevention measures when entering edge habitats where *A. americanum* proliferates. Furthermore, due to their proliferation in edge habitats, targeted interventions in these areas could reduce human–tick contact risk with *A. americanum*.

Contrary to our predictions, vegetation length was not a significant predictor, suggesting that adults may rely on the nearest available vegetation for host-seeking. When we tested models without temperature and humidity, no vegetation length category predicted tick abundance, suggesting that it is not the vegetation length itself, but the specific microclimatic conditions it creates that allow for tick survival. Combined with our spatial analysis results, these findings indicate that adult *A. americanum* can survive and host-seek across a broader range of habitats than nymphs. This contrasts with previous studies, which found that forest-related variables were the strongest predictors across all life stages [14, 51]. A previous study by Marshall et al. [26] found that adult *A. americanum* could travel up to 9 m in the field, supporting the idea that adults aggressively host-seek beyond forested habitats, possibly detaching from vertebrate hosts in these areas. Interestingly, proximity to water sources was a strong predictor of *A. americanum* abundance for both adults and nymphs (Table 1). While vertebrate hosts require water for survival, the southeastern USA has abundant water sources, and no studies to date have demonstrated wildlife aggregation at water sources in eastern US forests. However, in an arid environment, mammalian hosts aggregate around water sources, which can promote parasite abundance [52]. Further studies are needed to determine whether water sources function as tick aggregation hotspots for *A. americanum* in eastern US landscapes, and whether this is driven by vertebrate host movement or other factors.

Our best-fit nymph model suggests an association between nymphs and forested habitats, with key variables including the presence of leaf litter, tree shading, exposed soil, and forest-edge ecotones (Table 1). Additionally, nymph abundance was high in our domicile habitat, particularly in areas transitioning into forested habitat. This abundance was due to a single extreme outlier event in which 1033 nymphs were collected in a sampling area consisting of four adjacent squares (Fig. 1). Despite this, when we excluded outlier collection events, this area of the domicile continued to show elevated nymph counts, indicating a life stage-specific preference not seen in adults. The results of our hotspot analysis further support the conclusion that nymphs strongly prefer and appear to be restricted to forested habitats (Fig. 3). This finding parallels previous observations in the Lyme disease system, where *I. scapularis* nymphs demonstrate increased risk of encounters in forested environments [51, 53, 54]. While controlling ticks in forested environments at large scales is challenging, the increased risk of nymphal encounters can be communicated to encourage additional precautions in these areas, especially considering the higher risk of TBD transmission posed by nymphs compared to adults [55].

Our spatial analyses revealed significant clustering of infected ticks for both *Ehrlichia* spp. and *R. amblyommatis* (Figs. 4 and 5). Hotspots for both microbial agents were concentrated in forested and nearby forest-edge habitats where large numbers of nymphs were collected, suggesting that high nymphal densities are a significant risk factor for pathogen transmission (Figs. 3, 4, 5). This pattern aligns with findings from the Lyme disease system, where *I. scapularis* nymphs are considered the primary drivers of disease transmission [53, 56]. Additionally, pathogen hotspots generally corresponded to areas with the highest tick densities. Positive samples detected at all sampled habitat plots but primarily along habitat edges (Figs. 4 and 5). This was especially evident for *R. amblyommatis*, whose distribution closely mirrored overall tick collections (Fig. 5, Supplementary Figure 7), further supporting the hypothesis that this rickettsial species is an important endosymbiont of *A. americanum* [13, 57]. While our results indicate that disease risk is highest in forested habitats, the broader patterns suggest that pathogen risk is more closely associated with tick abundance than with habitat type. The presence of both microbial agents in all habitat plots supports this conclusion, which aligns with our expectations. Additionally, on one sampling day, five nymphal pools tested positive for all three bacteria, demonstrating the risk of co-infection with multiple human pathogens. It is currently unclear how these bacteria interact within tick vectors or with human immune responses. Given the potential risk of co-infection in the southeastern USA, future studies should investigate these interactions to better understand how co-infections shape epidemiological processes.

Our study did not assess vertebrate host activity, a critical factor, since ticks rely on vertebrate hosts to complete their life cycles. Because of this limitation, we were unable to directly link tick abundance to vertebrate activity. Although wildlife was abundant at our sampling site, we can only cautiously infer the role of vertebrates in shaping the distribution of *A. americanum*. Future studies should aim to clarify this relationship to better understand how vertebrates influence the fine-scale distribution of *A. americanum*. Furthermore, because our study was conducted intensively at a single site and lacked replication across multiple sampling sites, it remains unclear whether these patterns are consistent at the broader landscape scale. Future research should test our micro-landscape variables across multiple sites to determine whether they are widespread predictors of tick abundance or artifacts of site-specific sampling.

Ecological studies on ticks and their associated pathogens are valuable for identifying landscape characteristics linked to tick encounter risk, which can inform potential landowner recommendations for mitigating tick exposure. While mowing has been proposed as an effective tick control strategy [31], adult ticks were frequently collected in our SGF, indicating some exposure risk in these habitats even when mowed. However, nymphal ticks in our study, associated with the highest infection rates, were rarely collected outside of the edge ecotones in our SGF and LGF habitats. Combined with our observation that both adults and nymphs rely on more humid microclimates, mowing may significantly reduce overall tick encounters and subsequent disease transmission. The results of our model suggest that edge and forest habitats pose the highest risk for tick exposure at our sampling site, highlighting these areas as priorities for targeted chemical and physical interventions. One example of a physical intervention is exclusion fencing for white-tailed deer at domestic–sylvatic interfaces, which may be a promising strategy for reducing tick abundance [31] in peridomestic habitats, as deer are the primary host of this tick species [5, 6]. Ongoing research is exploring additional tick control methods, and future studies should incorporate these into ecological assessments to evaluate their impact on tick populations at the fine scales explored in our study.

We characterized the spatial distribution of *A. americanum* adults and nymphs, their associated microbial agents, and the environmental variables driving their presence within a sampling site comprised of diverse habitat types at a fine spatial scale. To our knowledge, this is the first study to assess the spatial distribution of this species at a meter-level resolution in conjunction with environmental risk assessments. These findings provide insights into a region experiencing significant increases in TBDs and population growth [58]. As this region experiences increased suburbanization and urban sprawl, mirroring the northeastern USA during the Lyme disease emergence in the previous century, we can expect increased exposures to *A. americanum* and its associated microbial agents. Furthermore, the results of this study may inform public health recommendations for landscape modifications to manage ticks and identify strategies to reduce the risk of tick exposure in the southeastern USA. For example, one recommendation is to take extra precautions against *A. americanum* nymphs (e.g., light-colored clothing treated with permethrin, tick checks after exposures) while in forested areas, particularly on forest edges. Specific recommendations like this will help combat the rise of *A. americanum*-associated pathogens in the southeastern USA.

## Conclusions

Our study provides novel insights into the fine-scale environmental factors influencing the distribution of *A. americanum* and associated microbial agents, including *Ehrlichia* spp. and *R. amblyommatis*, in the southeastern United States. By applying ultrafine-scale spatial and modeling approaches, an area of research previously understudied for *A. americanum*, we identified key habitat features correlated with tick presence and pathogen prevalence. This fine-scale focus addresses a critical gap in tick ecology where most research relies on broader spatial scales that may overlook important microhabitat influences. Given the public health significance of *A. americanum* and its pathogens, these findings have important implications for risk assessment, surveillance, and targeted control strategies. Ultimately, our work contributes valuable data and a refined methodological framework for understanding and mitigating TBD risk in this region.

## Supplementary Information

Supplementary Material 1.

Supplementary Material 2.

## Data Availability

Data supporting the main conclusions of this study are included in the manuscript.
