# Supplementary material for: Environmental correlates and fine-scale distribution of Amblyomma americanum, Ehrlichia spp., and Rickettsia amblyommatis at a single site in south-central Virginia
Source: Parasit Vectors. 2025 Sep 29;18:393. doi: 10.1186/s13071-025-06999-2 (PMC12481979; doi:10.1186/s13071-025-06999-2)
Supplement: Supplementary file 1 — Supplementary Material 1. [file 13071_2025_6999_MOESM1_ESM.docx]

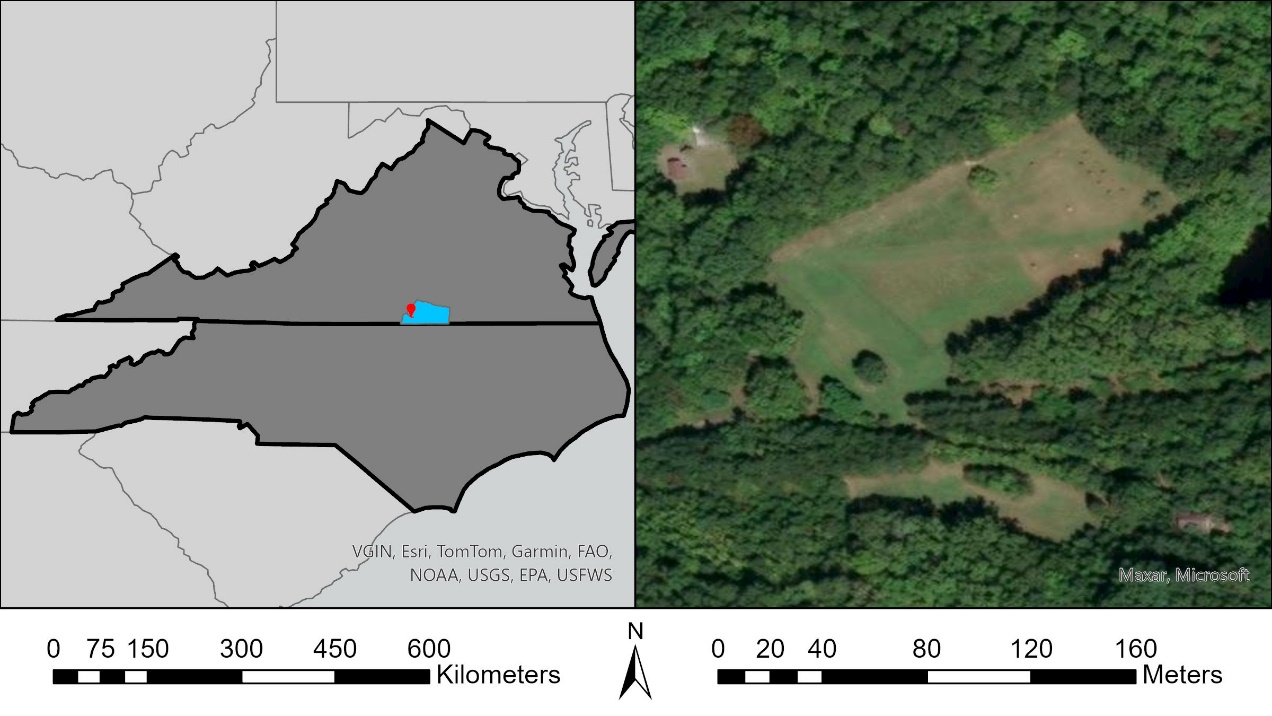


**Supplemental Figure 1.** A map showing the site location within Mecklenburg County, Virginia. The highlighted region (left) represents Mecklenburg County, and the point indicates the sampling site.


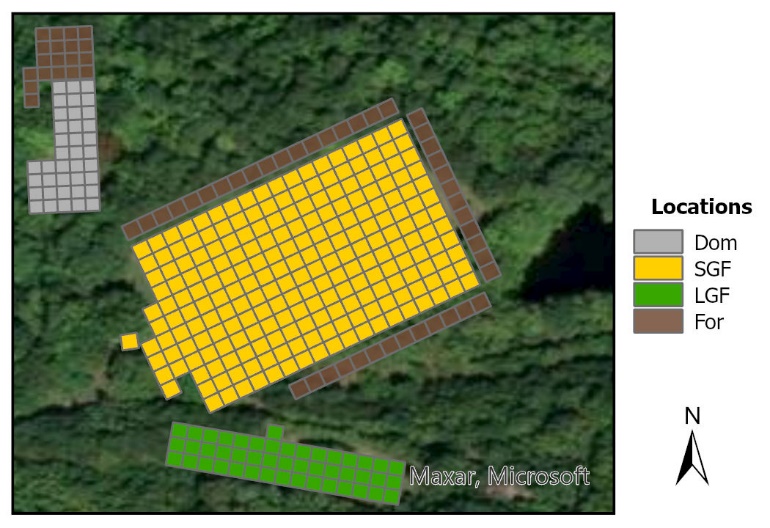


**Supplemental Figure 2**. Sampling distribution of habitats at the field site. Legend acronyms: Long Grass Field (LGF), Short Grass Field (SGF), Domicile (Dom), and Forest (For).


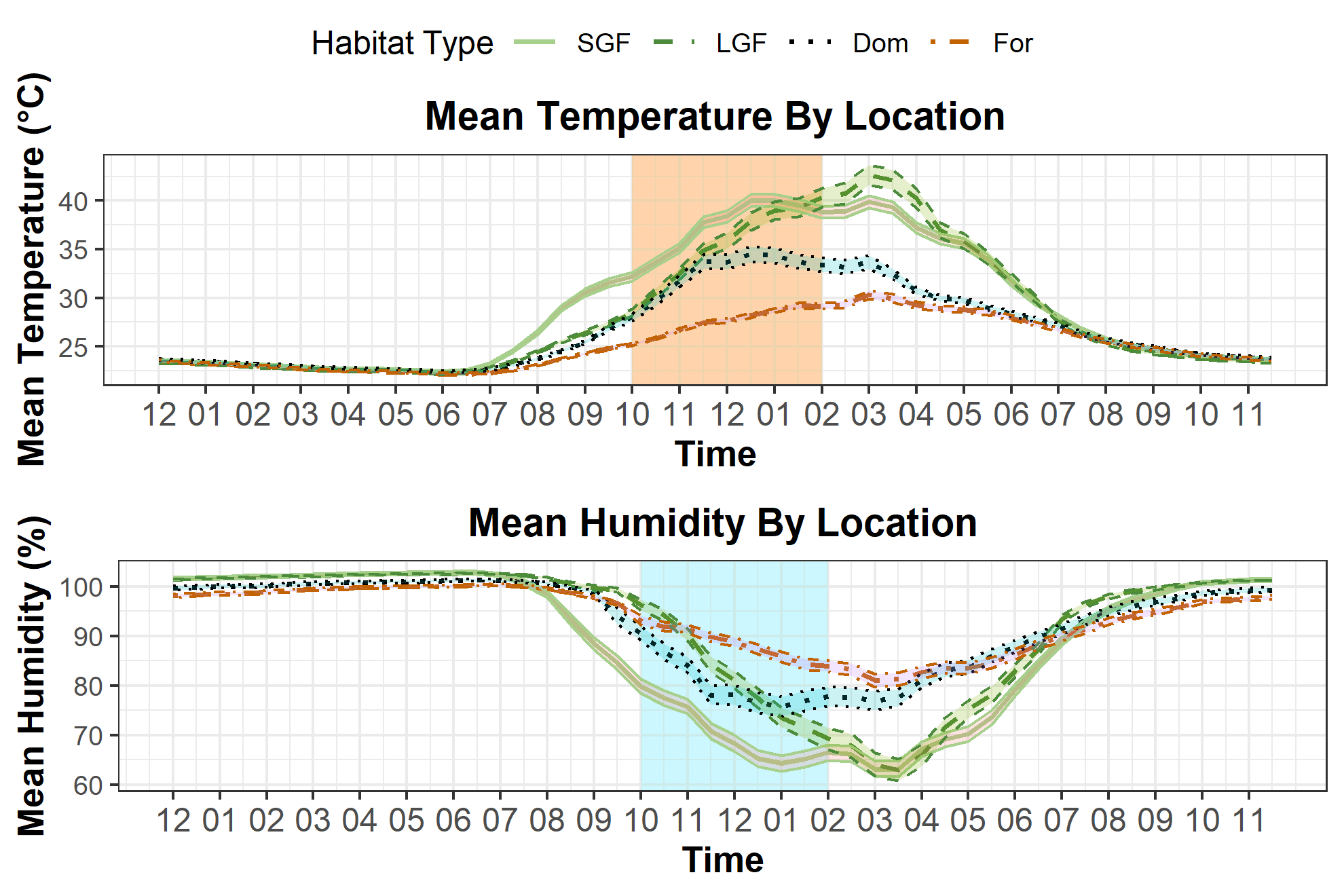


**Supplemental Figure 3.** Mean temperature (top) and mean relative humidity (bottom) across sampled habitat types over time (in hours). The shaded rectangle represents the sampling period (10 AM to 2 PM), when tick activity is expected to peak. Standard error is represented by the shaded region above and below each location line. Legend acronyms: Long Grass Field (LGF), Short Grass Field (SGF), Domicile (Dom), and Forest (For).


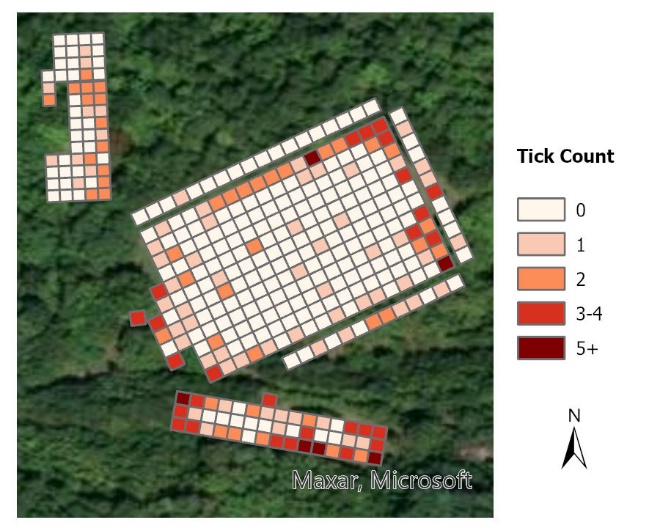


**Supplementary Figure 4.** Spatial distribution of *A. americanum* adult counts at the field site.


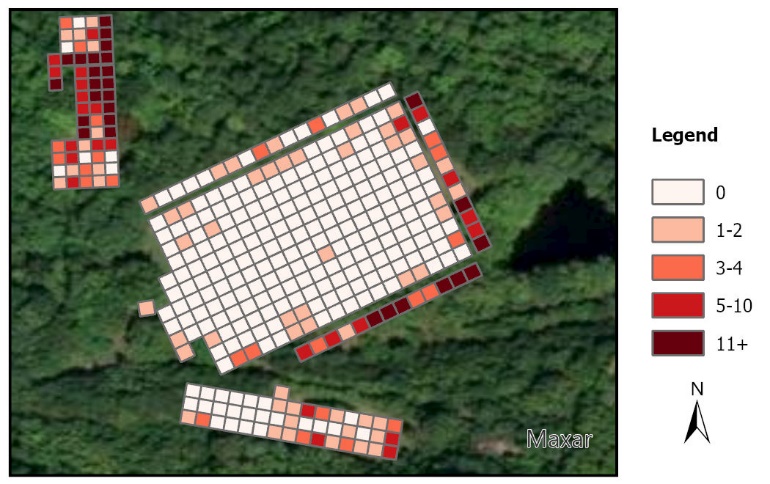


**Supplementary Figure 5.** Spatial distribution of *A. americanum* nymph counts at the field site.

**
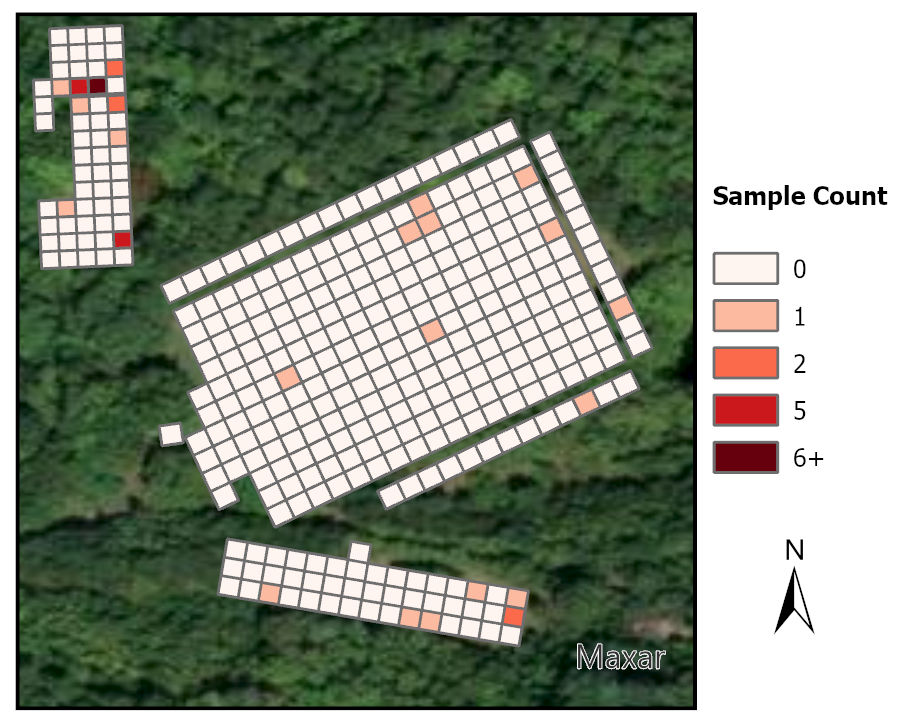
**

**Supplementary Figure 6.** Spatial distribution of *Ehrlichia*-positive *A. americanum* sample counts at the field site.


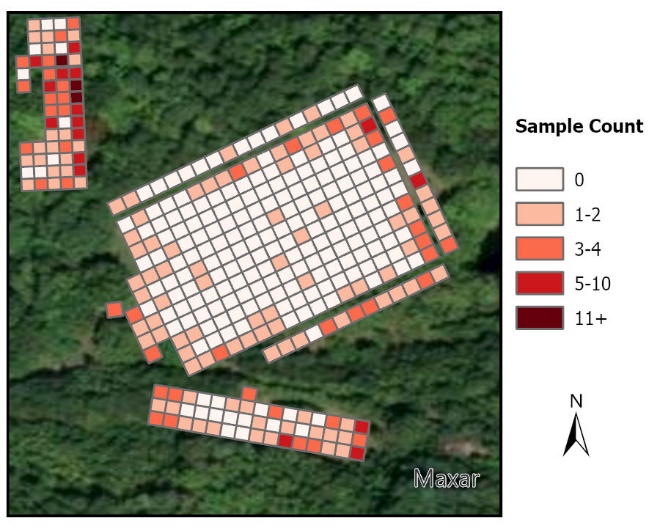


**Supplementary Figure 7**. Spatial distribution of *R. amblyommatis*-infected *A. americanum* sample counts at the field site.
