# Supplementary material for: Environmental correlates and fine-scale distribution of Amblyomma americanum, Ehrlichia spp., and Rickettsia amblyommatis at a single site in south-central Virginia
Source: Parasit Vectors. 2025 Sep 29;18:393. doi: 10.1186/s13071-025-06999-2 (PMC12481979; doi:10.1186/s13071-025-06999-2)
Supplement: Supplementary file 2 — Supplementary Material 2. [file 13071_2025_6999_MOESM2_ESM.docx]

| **Variable Name** | **Variable Type** |
| --- | --- |
| Temperature | Continuous |
| Humidity | Continuous |
| Order of Sampling | Continuous |
| Year | Categorical |
| Sampling Day | Continuous |
| Sampling Method | Categorical |
| Habitat Plot Type | Categorical |
| Short Vegetation | Binary |
| Medium Vegetation | Binary |
| Long Vegetation | Binary |
| Sparse Vegetation | Binary |
| Planted Ornamental Vegetation | Binary |
| Dried or Desiccated Vegetation | Binary |
| Landscaping Rocks | Binary |
| Leaf Litter Presence | Binary |
| Adjacency to Forest | Binary |
| Adjacency to Field | Binary |
| Adjacent to Road | Binary |
| Adjacency to Buildings or Structures | Binary |
| Water Source Proximity | Binary |
| Mowed Vegetation | Binary |
| Exposed Soil | Binary |

**Supplemental Table 1.** A list of all tested variables and their variable data types for generalized linear mixed modeling.

|  | **Year 1** | | | **Year 2** | | |
| --- | --- | --- | --- | --- | --- | --- |
| **Habitat** | **Adults** | **Nymphs** | **Effort (m^2^)** | **Adults** | **Nymphs** | **Effort** |
| LGF | 72 | 38 | 620 | 30 | 28 | 460 |
| SGF | 119 | 46 | 4510 | 15 | 8 | 2260 |
| Dom | 23 | 63 | 760 | 8 | 1312 | 380 |
| For | 4 | 128 | 110 | 20 | 347 | 620 |

**Supplemental Table 2.** Tick collection counts and sampling effort by year and habitat plot type. An additional Abbreviations: Long Grass Field (LGF), Short Grass Field (SGF), Domicile (Dom), and Forest (For). Effort is expressed as square meters sampled using dragging and flagging methods.

|  | **Year 1** | | | | **Year 2** | | | |
| --- | --- | --- | --- | --- | --- | --- | --- | --- |
|  | **Adults** | | **Nymphs** | | **Adults** | | **Nymphs** | |
| Habitat | Positive | Total | Positive | Total | Positive | Total | Positive | Total |
| LGF | 2 | 75 | 0 | 39 | 3 | 30 | 0 | 17 |
| SGF | 2 | 128 | 0 | 49 | 0 | 15 | 0 | 7 |
| Dom | 0 | 26 | 3 | 137 | 0 | 8 | 0 | 92 |
| For | 0 | 6 | 5 | 30 | 0 | 20 | 0 | 80 |

**Supplemental Table 3.** Number of *Ehrlichia ewingii*-positive pools by year, life stage, and habitat. Total pools tested for each are also provided. Abbreviations: Long Grass Field (LGF), Short Grass Field (SGF), Domicile (Dom), and Forest (For).

|  | **Year 1** | | | | **Year 2** | | | |
| --- | --- | --- | --- | --- | --- | --- | --- | --- |
|  | **Adults** | | **Nymphs** | | **Adults** | | **Nymphs** | |
| Habitat | Positive | Total | Positive | Total | Positive | Total | Positive | Total |
| LGF | 2 | 75 | 0 | 39 | 0 | 30 | 0 | 17 |
| SGF | 5 | 128 | 0 | 49 | 0 | 15 | 0 | 7 |
| Dom | 1 | 26 | 8 | 137 | 1 | 8 | 0 | 92 |
| For | 0 | 6 | 11 | 19 | 0 | 20 | 2 | 80 |

**Supplemental Table 4.** Number of *Ehrlichia chaffeensis*-positive pools by year, life stage, and habitat. Total pools tested for each are also provided. Abbreviations: Long Grass Field (LGF), Short Grass Field (SGF), Domicile (Dom), and Forest (For).

|  | **Year 1** | | | | **Year 2** | | | |
| --- | --- | --- | --- | --- | --- | --- | --- | --- |
|  | **Adults** | | **Nymphs** | | **Adults** | | **Nymphs** | |
| Habitat | Positive | Total | Positive | Total | Positive | Total | Positive | Total |
| LGF | 29 | 75 | 22 | 39 | 23 | 30 | 10 | 17 |
| SGF | 88 | 128 | 23 | 49 | 9 | 15 | 6 | 7 |
| Dom | 19 | 26 | 64 | 137 | 4 | 8 | 81 | 92 |
| For | 4 | 6 | 24 | 30 | 17 | 20 | 54 | 80 |

**Supplemental Table 5.** Number of *Rickettsia amblyommatis-*positive pools by year, life stage, and habitat. Total pools tested for each are also provided. Abbreviations: Long Grass Field (LGF), Short Grass Field (SGF), Domicile (Dom), and Forest (For).
